# Draft genome sequence of *Legionella lytica* DSB2004 isolated from a fire sprinkler system

**DOI:** 10.1128/mra.01256-24

**Published:** 2025-06-30

**Authors:** Alyssa D. Everhart, Destaalem T. Kidane, Anthony L. Farone, Mary B. Farone

**Affiliations:** 1 Department of Biology, Middle Tennessee State University 171746, Murfreesboro, Tennessee, USA; 2 Molecular Biosciences Program, Middle Tennessee State University 5235, Murfreesboro, Tennessee, USA; SUNY College of Environmental Science and Forestry, Syracuse, New York, USA

**Keywords:** *Legionella*, amoeba, flagella, motility, intracellular

## Abstract

We present the draft genome sequence of *Legionella lytica* strain DSB2004 isolated from a fire sprinkler system. The size of the genome is 3,983,660 bp, consisting of 3,626 coding sequences and a GC content of 40.3%. Genomic analysis identified genes encoding a type IV secretion system and proteins for flagellar motility.

## ANNOUNCEMENT

*Legionella* spp. are gram-negative coccobacilli commonly found in natural aquatic environments; however, *Legionella* spp. also thrive in artificial environments such as cooling towers and air-conditioning systems (1). *Legionella lytica*, formerly known as *Sarcobium lyticum*, was first isolated from a free-living amoeba (FLA) recovered from a soil sample in Poland (2–4). Since its isolation, *L. lytica* has been shown to infect and replicate in several species of FLA, including *Acanthamoeba castellanii* and *Hartmannella astronyxis* (3). Infection and replication of this bacterium in human monocytes, as well as detection of antibodies against *L. lytica* in pneumonia patients, have implicated *L. lytica* in human illnesses (5).

This *L. lytica* isolate was recovered from biofilm collected from the fire sprinkler system of a school building in Murfreesboro, Tennessee, USA. Sterile swabs were used to sample the biofilm of existing plumbing during the installation of new pipes. Swabs were rolled onto the centers of non-nutrient agar plates cross-streaked with heat-killed *Escherichia coli* (ATCC 25922). Plates were incubated at 25°C and observed microscopically for amoebae with intracellular bacteria. If observed, agar plugs containing infected amoebae were added to *Acanthamoeba polyphaga* (ATCC 30461) monolayers, and these co-cultures were monitored for amoeba infection. The bacterium was isolated from amoebae by growth on BD BBL BCYE agar at 25°C but was maintained in *A. polyphaga* co-culture (6). This bacterium was of interest due to its rapid motility in amoebae. *L. lytica* were separated from amoebae using Renografin density-gradient centrifugation (7). Bacterial DNA was extracted using the MasterPure Complete DNA Purification Kit (Lucigen) following the manufacturer’s guidelines.

Library preparation and genome sequencing were performed at Novogene Inc. The NEBNext Ultra II DNA library kit (E7645, New England Biolabs) was used for library preparation. Library quality was assessed using an Agilent 2100 Bioanalyzer (Agilent Technologies) for size distribution. Genome sequencing was performed using the Illumina MiSeq sequencing platform. Sequencing produced a total of 8,928,012 150-base pair (bp) paired-end reads.

The galaxy web-based platform was used for genome assembly (https://usegalaxy.org/). Sequence read data quality was assessed with FastQC (version 0.73). Reads were trimmed using Trimmomatic (Galaxy version 0.38.0). The Shovill pipeline, included with a SPAdes assembler (version 1.1.0), was used to assemble sequenced reads into contiguous sequences. The assembly was filtered, retaining contigs larger than 200 bp (Table 1). The genome was annotated using the Rapid Annotations using Subsystems Technology (RAST) server and Prokaryotic Genome Annotation Pipeline (PGAP, version 6.8) (8, 9).

**TABLE 1:** Genome features of *L. lytica* DSB2004

Feature	Data for *L. lytica* DSB2004
Number (No.) of contigs	49
N50 contig size (bp)	545,140
Largest contig (bp)	1,769,598
Total genome size (bp)	3,983,660
Genome coverage (x)	180
G + C content (%)	40.3
Mean Phred quality score	>36
No. of coding sequences	3,407
No. of rRNAs	3
No. of tRNAs	40
No. of protein-coding genes	3,310
No. of Type IVB secretion system genes	27
No. of antibiotic resistance genes^*a*^	3
No. of pseudogenes	46

^*a*^ Antibiotic resistance genes were determined using the Analyze function and BLAST search tool of the Comprehensive Antibiotic Resistance Database (CARD, version 4.0.0, https://card.mcmaster.ca/analyze) (10).

The genome was 3,983,660 bp with 40.3% GC content, 3,584 putative protein-coding sequences (45.42% hypothetical genes), and 43 RNA-coding sequences (Table 1). Based on the 16S rRNA gene sequence, the bacterium was identified as *L. lytica* and, using the National Center for Biotechnology (NCBI) Basic Search Alignment Tool (BLAST), exhibited 99.35% similarity to *L. lytica* strain PCM 2298 (NCBI GenBank accession CP071527.1). Genome annotation with RAST identified dot/icm type IVB secretion system genes (Table 1), and notably, at least 58 genes encoding proteins for flagellar functions.

## Data Availability

The genome sequence file of *L. lytica* strain DSB2004 has been deposited at GenBank under accession number JBGORX000000000 (https://www.ncbi.nlm.nih.gov/nuccore/JBGORX000000000). PGAP annotations are available at https://www.ncbi.nlm.nih.gov/datasets/gene/GCA_044626755.1/. Raw sequencing reads have been deposited in the NCBI Sequence Read Archive under accession number SRR30497534 (https://trace.ncbi.nlm.nih.gov/Traces/?view=run_browser&page_size=10&acc=SRR30497534&display=reads).
